# Microbial metagenomic shifts in children with acute lymphoblastic leukaemia during induction therapy and predictive biomarkers for infection

**DOI:** 10.1186/s12941-024-00717-z

**Published:** 2024-06-15

**Authors:** Huidi Wang, Yajie Zhang, Qianyi Zhou, Lihua Yu, Jingxiang Fu, Danna Lin, Lulu Huang, Xiaorong Lai, Li Wu, Jingxin Zhang, Juan Zi, Xu Liao, Siying Huang, Yugu Xie, Yan He, Lihua Yang

**Affiliations:** 1 Microbiome Medicine Center, Department of Laboratory Medicine, Zhujiang Hospital, Southern Medical University, Guangzhou, 510280 Guangdong China; 2 Department of Paediatric Hematology, Zhujiang Hospital, Southern Medical University, Guangzhou, 510280 Guangdong China; 3 Department of Laboratory Medicine, Clinical Biobank Centre, Microbiome Medicine Center, Zhujiang Hospital, Southern Medical University, Guangzhou, 510280 China

**Keywords:** Acute lymphoblastic leukaemia, Gut microbiota, Infection, Children, Biomarker

## Abstract

**Background:**

Emerging evidence has indicated a link between the gut microbiota and acute lymphoblastic leukaemia (ALL). However, the acute changes in gut microbiota during chemotherapy and the predictive value of baseline gut microbiota in infectious complication remain largely unknown.

**Methods:**

Faecal samples (*n* = 126) from children with ALL (*n* = 49) undergoing induction chemotherapy were collected at three timepoints, i.e., initiation of chemotherapy (baseline, T0), 7 days (T1) and 33 days (T2) after initiation of chemotherapy. Gut microbiome profile was performed via metagenomic shotgun sequencing. The bioBakery3 pipeline (Kneaddata, Metaphlan 3 and HUMAnN) was performed to assign taxonomy and functional annotations. Gut microbiome at T0 were used to predict infection during chemotherapy.

**Results:**

The microbial diversities and composition changed significantly during chemotherapy, with *Escherichia coli*, *Klebsiella pneumoniae* and *Bifidobacterium longum* being the most prominent species. The microbial metabolic pathways were also significantly altered during chemotherapy, including the pathway of pyruvate fermentation to acetate and lactate, and assimilatory sulfate reduction pathway. The receiver operating characteristic (ROC) models based on *Bifidobacterium longum* at T0 could predict infectious complications during the first month of chemotherapy with the area under the curve (AUC) of 0.720.

**Conclusions:**

Our study provides new insights into the acute changes in microbial and functional characteristics in children with ALL during chemotherapy. The baseline gut microbiota could be potential biomarkers for infections during chemotherapy.

**Trial registration:**

The study was approved by the Ethics Committee of Zhujiang Hospital, Southern Medical University (2021-KY-171-01) and registered on http://www.chictr.org.cn (ChiCTR2200065406, Registration Date: November 4, 2022).

**Supplementary Information:**

The online version contains supplementary material available at 10.1186/s12941-024-00717-z.

## Introduction

Acute lymphoblastic leukaemia (ALL) is a malignant neoplastic disease of the bone marrow characterised by excessive proliferation and accumulation of immature lymphocytes which can spread to extramedullary sites. Leukaemia accounts for the most common cancer in children, and ALL represents the main subtype of leukaemia, with the proportion ranging from approximately 60–80% in children under the age of 15 years [1]. Chemotherapy is the primary treatment for ALL, which includes three phases, i.e., the induction, consolidation, and maintenance phase [2]. Unfortunately, patients undergoing chemotherapy often experience immunosuppression and subsequent infectious complication. Bacterial infection is the major cause of morbidity and mortality in children with ALL and children are most susceptible during the induction phase [3]. Previously, extensive researches have focused on long-term prognostic factors to evaluate the risk of disease relapse [4]. However, studies investigating short-term complications such as infection during chemotherapy remain scarce and require further investigation.

Over the years, the research of gut microbiota has gained more and more appreciation due to its extensive connections with human health [5, 6]. Although the aetiology of ALL remains inconclusive, the genetic and environmental factors are widely acknowledged as the key factors that contribute to the development and progression of ALL [7]. More importantly, these two factors are strongly associated with the gut microbiota. A recent study proposes that immaturity of microbiome in early life may participate in the pathogenesis of ALL and warrants future microbiota-targeted intervention [8]. In terms of the gut microbiota during chemotherapy, there is an increasing need to understand the impact of gut microbiota on the efficacy and complication of chemotherapy. It is of certainty that chemotherapeutic drugs would have great impact on the composition of the gut microbiota. On one hand, chemotherapy can directly damage healthy intestinal cells, leading to gastrointestinal disturbance such as diarrhea, vomiting and abdominal pain, which indirectly affects microbial ecosystem [9]. The use of prophylactic or therapeutic broad-spectrum antibiotics further disrupts the balance of gut ecosystem. On the other hand, the dysbiotic gut microbiota may affect the metabolism and absorption of the chemotherapeutic chemicals, augmenting the toxicity and diminishing the efficacy of the drugs. The interactions between chemotherapeutic drug, gastrointestinal tract and gut microbiota lead to impaired gut mucosal barrier integrity, facilitating translocation of bacteria that are affected by chemotherapeutic drugs, and further increase the risk of infectious complication.

The gut microbiota can be easily accessible and uniquely modifiable, providing a potential to improve gut dysbiosis of ALL. Improved personalized use of probiotics, prebiotics and antibiotics based on microbial markers to reduce the risk of infection may be of great interest to paediatricians and, more importantly, beneficial to patients with ALL. Hence, there is an urgent need to understand the changes of the gut microbiota during chemotherapy and to identify microbial biomarkers that can predict infectious complication following initiation of chemotherapy. In the present study, using a cohort of children with ALL, we investigated the dynamic changes of gut microbiota and related microbial metabolic pathways during induction chemotherapy at three different timepoints, i.e., initiation of chemotherapy (baseline, T0), 7 days (T1) and 33 days (T2) after initiation of chemotherapy. We also analysed the potential microbial biomarkers at T0 to predict infectious complication during chemotherapy. Our study provides new insights into the dynamic changes in microbial and metabolic characteristics in children with ALL during chemotherapy and could help to discover microbiota-targeted intervention to prevent infectious complication during chemotherapy.

## Materials and methods

### Ethics statement

The study was approved by the Ethics Committee of Zhujiang Hospital, Southern Medical University (2021-KY-171-01) and registered on http://www.chictr.org.cn (ChiCTR2200065406, Registration Date: November 4, 2022). Written informed consent was obtained from all parents in compliance with the Declaration of Helsinki.

### Study design

The subjects were recruited from the Department of paediatrics of Zhujiang Hospital of Southern Medical University (Guangzhou, China) from January 2022 to May 2023. The inclusion criteria were as follows: (i) an age ranging from 1 to 15 years, (ii) diagnosis of ALL according to the morphological, immunological, cytogenetic and molecular (MICM) standards, (iii) first-episode children. The exclusion criteria were as follows: (i) patients who had previously received chemotherapy or other anticancer therapies such as immunotherapy, radiotherapy or hematopoietic stem cell transplantation (HSCT) at the time of admission, (ii) history of other types of cancer and (iii) history of glucocorticoid use within 1 month before enrolment, and (iv) ineligible for chemotherapy. All patients received the standard chemotherapy according to the South China Children’s Leukaemia Group-ALL-2016 (SCCLG-ALL-2016) protocol [10]. According to the protocol, the treatment commences with evaluation of sensitivity after 7 days of pretreatment with prednisone, followed by initiation of VDLD (vincristine + dexamethasone + L-asparaginase + daunorubicin) induced remission therapy, and early intensive CAM (cyclophosphamide + cytarabine + 6-mercaptopurine), mM (methotrexate + 6-mercaptopurine, or high risk (HR)-1 (dexamethasone + vincristine + methotrexate + cyclophosphamide + cytarabine + PEG-asparaginase), HR-2 (dexamethasone + vindesine + methotrexate + ifosfamide + daunorubicin + PEG-asparaginase), HR-3 (dexamethasone + cytarabine + daunorubicin + PEG-asparaginase) all in two rounds), delayed intensive VDLD, CAM regimen (with 8 weeks of maintenance chemotherapy in between) in one or two rounds, and finally maintenance chemotherapy and regular intrathecal injections. During the VDLD induction therapy (T0-T2), patients were hospitalized and we only looked for infectious complications during induction. Infectious complication was defined as any clinically or microbially-defined infection and/or clinically-documented febrile neutropenia event. We did not exclude patients who had received antibiotics before diagnosis or enrolment because of a high proportion of such patients [11]. 

### Risk stratification

ALL risk level was assessed according to the SCCLG-ALL-2016 protocol, taking into account the clinical characteristics, cellular immunology, biological characteristics, and treatment response of patients. After 7 days of pretreatment with prednisone, peripheral blast cells < 1.0 × 10^9^/L was considered prednisone good response (PGR), peripheral blast cells > 1.0 × 10^9^/L was considered prednisone poor response (PPR). Low risk (LR): (i) patients with PGR, (ii) an age ≥ 1 and < 10 years, (iii) peripheral white blood cell (WBC) count < 50 × 10^9^/L, (iv) bone marrow M1 (blast cells < 5%) on day 15 of induction chemotherapy, (v) bone marrow M1 on day 33 of induction chemotherapy. Patients who meet all the standards above were considered LR. Intermediate risk (IR): (i) an age < 1 year or ≥ 10 year, (ii) WBC count ≥ 50 × 10^9^/L, (iii) diagnosed with T-ALL, (iv) stage 3 of central nervous system (CNS3, defined by cerebrospinal fluid (CSF) WBC > 5/µl with blast cells in non-traumatic lumbar puncture (TLP), or CSF WBC > 5/µl and the CSF leukaemia cells proportion higher than peripheral blood naïve cells proportion (CSF WBC/red blood cell (RBC) ≥ 2 × peripheral WBC/RBC) in TLP, or any clinical or imaging (CT/MRI) evidence of CNS leukaemia regardless of CSF results) or diagnosed with testicular leukaemia, (v) Philadelphia chromosome (Ph)-positive ALL or Ph-like ALL defined by PCR test of the bone marrow, (vi) E2A-PBX1 fusion gene status, (vii) BCR-ABL1 fusion gene status, (viii) bone marrow M2 (blast cells 5% to < 25%) on day 15 of induction chemotherapy and bone marrow M1 on day 33 of induction chemotherapy. Patients with PGR and meet one or more of the standards above were considered IR. High risk (HR): (i) patients with PPR, (ii) bone marrow M3 (blast cells ≥ 25%) on day 15 of induction chemotherapy, (iii) bone marrow M2 or M3 on day 33 of induction chemotherapy, (iv) MLL-AF4 or other MLL rearrangement of bone marrow, (v) hypodiploid (chromosome number < 44), (vi) iAMP21 positive in bone marrow, (vii) IKZF1 deletion mutation, (viii) MEF2D or ZNF384 rearrangement, (ix) TCF3-HLF fusion gene status, (x) the mediastinal tumour did not shrink to one-third of the original tumour volume on day 33 of induction chemotherapy or the tumour was still present before consolidation therapy. Patients who meet one or more of the standards above were consider HR.

### Sample collection

Faecal samples were collected from each patient at three predefined timepoints, i.e., prior to initiation of chemotherapy (baseline, T0), 7 days (T1) and 33 days (T2) after initiation of chemotherapy. All the faecal samples were collected during hospitalization. Fresh faecal samples were collected from each patient using sterilized vials and put into liquid nitrogen and then delivered to laboratory and stored at -80 ℃ in refrigerator. A total of 18 samples were missing because the patients did not have the desire to defaecate at the predefined dates of sample collection.

### DNA extraction, sequencing, and bioinformatics

Genomic DNA was extracted by Guangdong Magigene Biotechnology Co.,Ltd. (Guangzhou, China) using commercial kits according to the manufacturer’s instructions. DNA integrity and purity were monitored on 1% agarose gels. DNA concentration and purity were measured using Qubit 3.0 (Thermo Fisher Scientific, Waltham, USA) and Nanodrop One (Thermo Fisher Scientific, Waltham, USA) at the same time. Sequencing libraries were generated using ALFA-SEQ DNA Library Prep Kit following manufacturer’s recommendations and index codes were added. The library quality was assessed on the Qubit 4.0 Fluorometer (Life Technologies, Grand Island, NY) and Qsep400 High-Throughput Nucleic Acid Protein Analysis system (Houze Biological Technology Co, Hangzhou, China) system. At last, the library was sequenced on an Illumina NovaSeq 6000 platform and 150 bp paired-end reads were generated. Sequences were then processed using KneadData pipeline with Trimmomatic [12] and Bowtie 2 [13] to remove low-quality reads and contaminates. Sequences were trimmed using Trimmomatic with parameters ILLUMINACLIP: TruSeq3-PE.fa:2:30:10 SLIDINGWINDOW:5:20 MINLEN:36 LEADING:3 TRAILING:3, and host sequences were removed using Bowtie 2 with default parameters. For taxonomic annotations, MetaPhlAn3 [14] was used for taxonomic profiling and quantification of relative abundances of organisms in all samples. For functional annotations, HUMAnN3 [14] was used with uniref90 as the protein database and Chocophlan as the nucleotide database, and the unstratified output of path abundance with MetaCyc [15] annotations was utilized in the analysis.

### Statistical analysis

Wilcoxon rank-sum test was utilized to calculate the α-diversity index between groups. Principal coordinate analysis (PCoA) was performed using Quantitative Insights Into Microbial Ecology (QIIME) modules. Linear discriminant analysis effect size (LEfSe) utilized the non-parametric Kruskal-Wallis rank-sum test to compare relative abundances of all bacterial taxa or functional features to compare the discriminative data between groups [16], and a linear discriminant analysis (LDA) value was calculated to represent the difference in the feature between groups. LDA score > 2.0 were included in the plot.

## Results

### Characteristics of participants

The demographic characteristics of participants were summarized in Table 1. A total of 49 children with ALL were enrolled in this study, including 28 males and 21 females. The median age was 5.2, ranging from 1 to 14. Twenty-nine (59%) patients were complicated with infection during VDLD induction chemotherapy. All the patients with infectious complication were diagnosed between T1 to T2, with 10 patients suffering from bacterial infection, 4 patients suffering from viral infection and 1 patient suffering from fungal infection.

**Table 1:** Clinical characteristics of the children with ALL (*n* = 49)

Characteristic	No. (%)
Age at diagnosis, y, median (range)	5.3 (1–14)
Age at diagnosis, y, No. (%)	
1–4 5–9 ≥ 10	27 (55) 15 (31) 7 (14)
Sex, No. (%)	
Male	28 (57)
Female	21 (43)
ALL type, No. (%)	
T-ALL B-ALL	7 (14) 42 (86)
ALL risk level at induction, No. (%)	
Low	3 (6)
Medium	22 (45)
High	24 (49)
Infectious complication, No. (%)	
Yes	29 (59)
No	20 (41)
Timing of onset of infectious complications, No. (%)	
T0 to T1	0 (0)
T1 to T2	29 (100)
Documented pathogen, No. (%)	15 (31)
Escherichia coli	1 (2)
Klebsiella pneumoniae Pseudomonas aeruginosa	2 (4) 2 (4)
Salmonella Pyogenic streptococcus Staphylococcus aureus Haemophilus influenzae and Streptococcus pneumoniae Rotavirus Human polyoma virus Human herpesvirus 1 Non-Candida albicans	2 (4) 1 (2) 1 (2) 1 (2) 2 (4) 1 (2) 1 (2) 1 (2)

### The microbial diversity changed drastically during induction chemotherapy

**Fig. 1:**
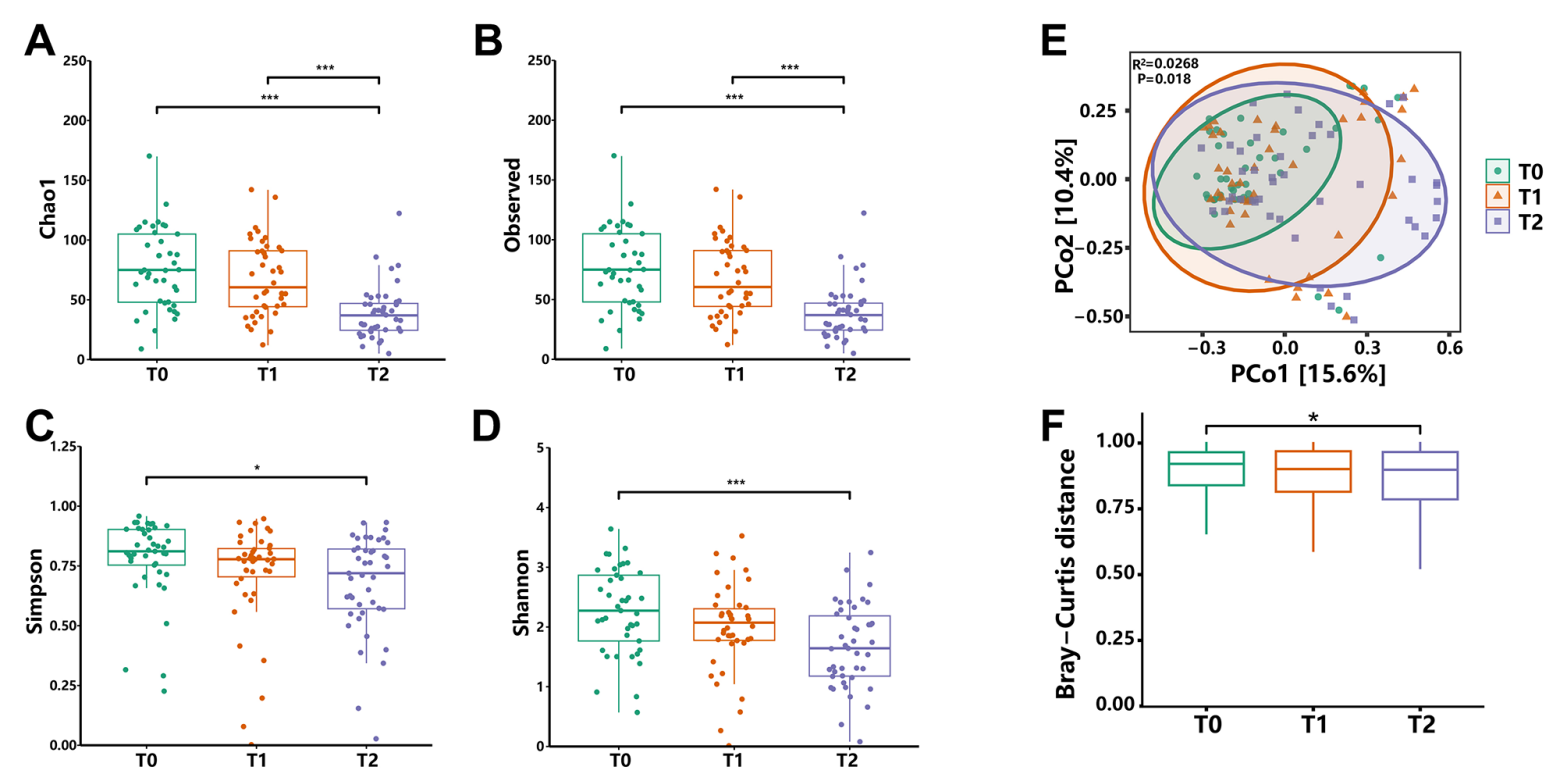
The α- and β-diversity of gut microbiota in children with ALL during the induction chemotherapy. The α-diversity indices measure the total amount of different species (richness) and/or how balanced the relative abundance of species are (evenness) in a microbial ecosystem (within a single sample). Comparison of the **(A)** Chao1 index (indicates richness), **(B)** Observed species (indicates richness), **(C)** Simpson index (indicates evenness), and **(D)** Shannon index (indicates both richness and evenness). The β-diversity calculates the degree of similarity and distance based on phylogenetic trees and measures the difference between the composition of two microbial communities. **(E)** The Bray-Curtis dissimilarity matrix and PERMANOVA were performed to measure the gradual shifts in the overall compositions of the gut microbiota. **(F)** The Bray-Curtis distance was calculated to investigate the extent of microbial changes. Each point represents a sample. Data are represented as mean ± SD, *p < 0.05, **p < 0.01, ***p < 0.001

To investigate the overall shifts of the gut microbiota during induction chemotherapy, we first measured the α- and β-diversity. The α-diversity indices measure the total amount of different species (richness) and/or how balanced the relative abundance of species are (evenness) in a microbial ecosystem (within a single sample). As is shown in Fig. 1, the α-diversity continued to reduce during induction chemotherapy. Specifically, the Chao1 index (indicates richness) and Observed species (indicates richness) in T2 were significantly decreased compared to those in T0 and T1 (Fig. 1A, B). The Simpson index (indicates evenness) and Shannon index (indicates both richness and evenness) in T2 were significantly decreased compared to those in T0 (Fig. 1C, D). The β-diversity calculates the degree of similarity and distance based on phylogenetic trees and measures the difference between the composition of two microbial communities. As a result, the gut microbiota exhibited a gradual shift in composition during the induction chemotherapy (Fig. 1E). We calculated the microbial difference based on Bray-Curtis distance, a measurement that quantifies the level of dissimilarity between the composition of two microbial communities, we found a significantly changed microbiota composition between T0 and T2 (Fig. 1F).

### The microbial composition shifted remarkably during induction chemotherapy

**Fig. 2:**
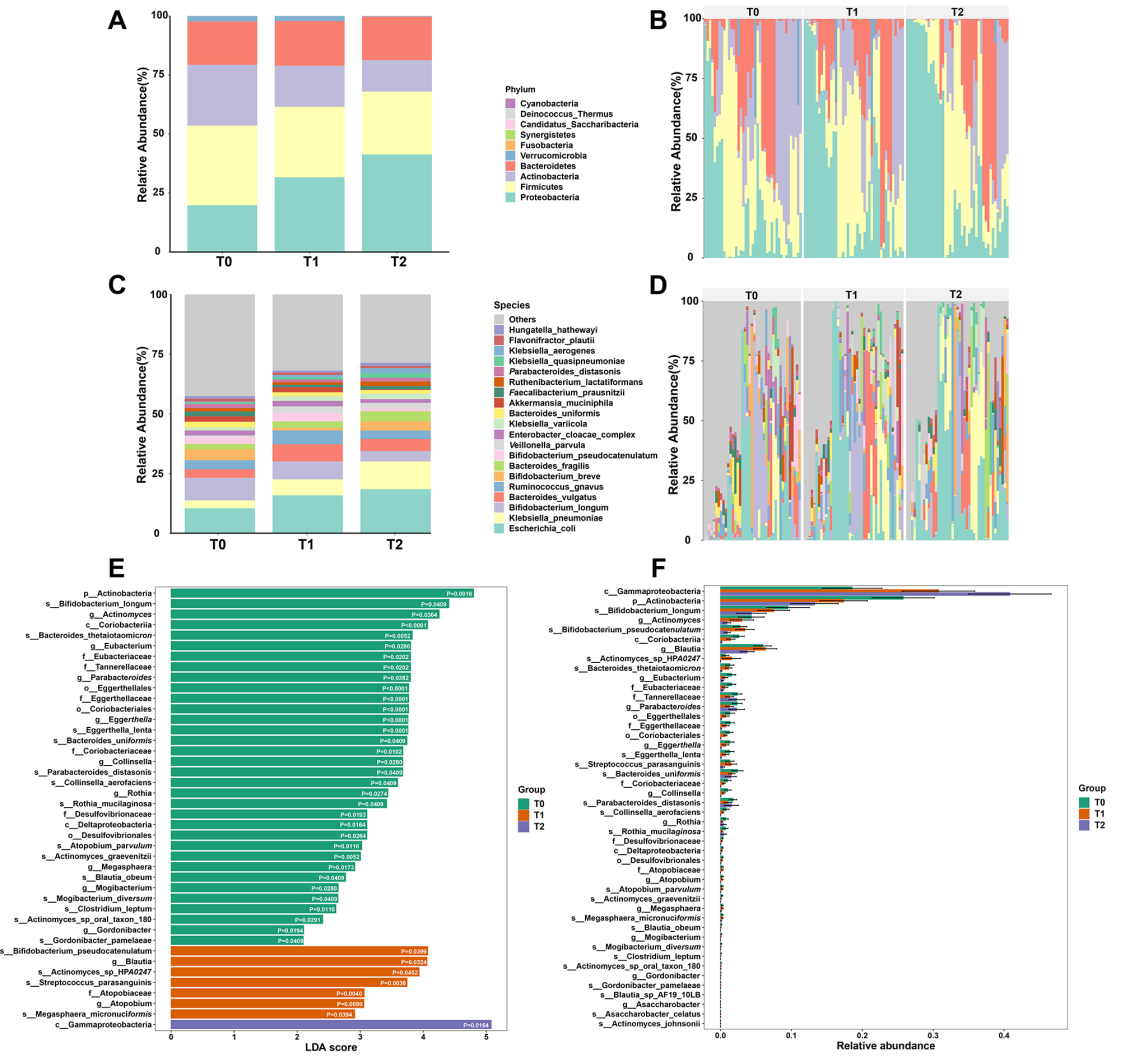
The gut microbial profile shifted remarkably during induction chemotherapy. (**A-B**) Relative abundances of gut microbiota at the phylum level in children with ALL during induction chemotherapy, clustered in groups **(A)** or individually **(B)**. **(C-D)** Relative abundances of gut microbiota at the species level in children with ALL during induction chemotherapy, clustered in groups **(C)** or individually **(D)**. **(E)** The microbiota that are significantly different in relative abundance and biological significance based on LEfSe analysis. **(F)** The relative abundances of microbiota that are significantly different between different timepoints. Data are represented as mean ± SD

To further explore the specific microbial changes during chemotherapy, we analysed the microbiota composition at different taxonomic levels. At the phylum level, the relative abundance of *Actinobacteria* decreased over time while the abundance of *Proteobacteria* increased during induction chemotherapy (Fig. 2A, B). At the species level, the relative abundances of *Escherichia coli*, *Klebsiella pneumoniae* and *Bacteroides fragilis* increased while the abundance of *Bifidobacterium longum* continued to decrease during induction chemotherapy (Fig. 2C, D). To identify the bacterial species that exhibit difference in relative abundance and biological significance, we used LEfSe analysis and found that *Actinobacteria* (*p* = 0.0016), *Bifidobacterium longum* (*p* = 0.0409), and *Actinomyces* (*p* = 0.0364) were enriched with the highest LDA score in T0; *Bifidobacterium pseudocatenulatum* (*p* = 0.0399) and *Blautia* (*p* = 0.0324) were enriched with the highest LDA score in T1; and *Gammaproteobacteria* (*p* = 0.0164) was enriched with the highest LDA score in T2 (Fig. 2E, F). The relative abundances of microbiota in other taxonomic levels are shown in Supplementary Fig. 1. In the three participants who were infected with documented pathogens detected from the blood (Table 1), including *Escherichia coli* and *Klebsiella pneumoniae*, the relative abundances of corresponding gut microbiota were extremely high (about 20% and 40%, data not shown) in these participants, suggesting that the pathogens were originated from the gut.

### The changes in microbial functional pathways during induction chemotherapy

**Fig. 3:**
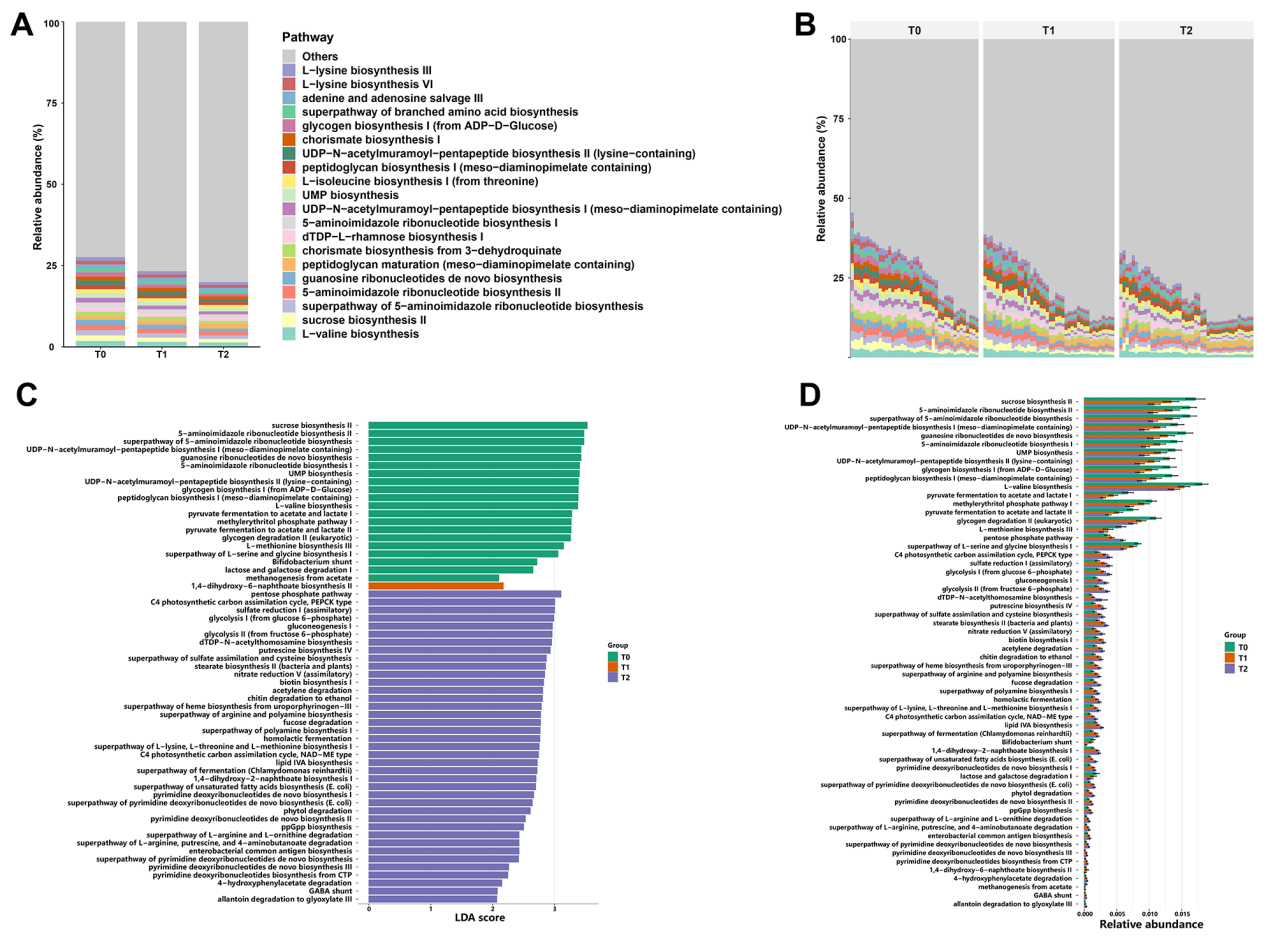
The shifts in microbial functional pathways during induction chemotherapy. (**A-B**) Relative abundances of microbial functional pathways at the three timepoints during induction chemotherapy, clustered in groups **(A)** or individually **(B)**. **(C)** The microbial functional pathways that are significantly different between the three timepoints based on LEfSe analysis. **(D)** The relative abundances of microbial functional pathways that are significantly different between the three timepoints. Data are represented as mean ± SD

To further investigate the biological significance of the gut microbiota during induction chemotherapy, we used HUMAnN 3.0 to analyse the abundance of functional pathways from metagenomic data. The most abundant microbial pathways include (i) the L-valine biosynthesis, (ii) sucrose biosynthesis II, and (iii) super pathway of 5-aminoimidazole ribonucleotide biosynthesis (Fig. 3A, B). The most abundant pathways from superclass 1 and superclass 2 are biosynthesis and amino acid biosynthesis, respectively (Supplementary Fig. S2A-D). We also performed LEfSe analysis and found that the sucrose biosynthesis II and 5-aminoimidazole ribonucleotide biosynthesis II were downregulated at T2, while the pentose phosphate pathway, C4 photosynthetic carbon assimilation cycle, and sulfate induction I (assimilatory) were upregulated (Fig. 3C, D). At superclass 1, the biosynthesis pathway was downregulated while the generation of precursor metabolites and energy was upregulated at T2 (Supplementary Fig. S2E, F). At superclass 2, the amino acid biosynthesis was downregulated while the fatty acid and lipid biosynthesis was upregulated at T2 (Supplementary Fig. S2G, H).

### Relative abundance of *Bifidobacterium longum* at baseline can predict infection during induction chemotherapy

**Fig. 4:**
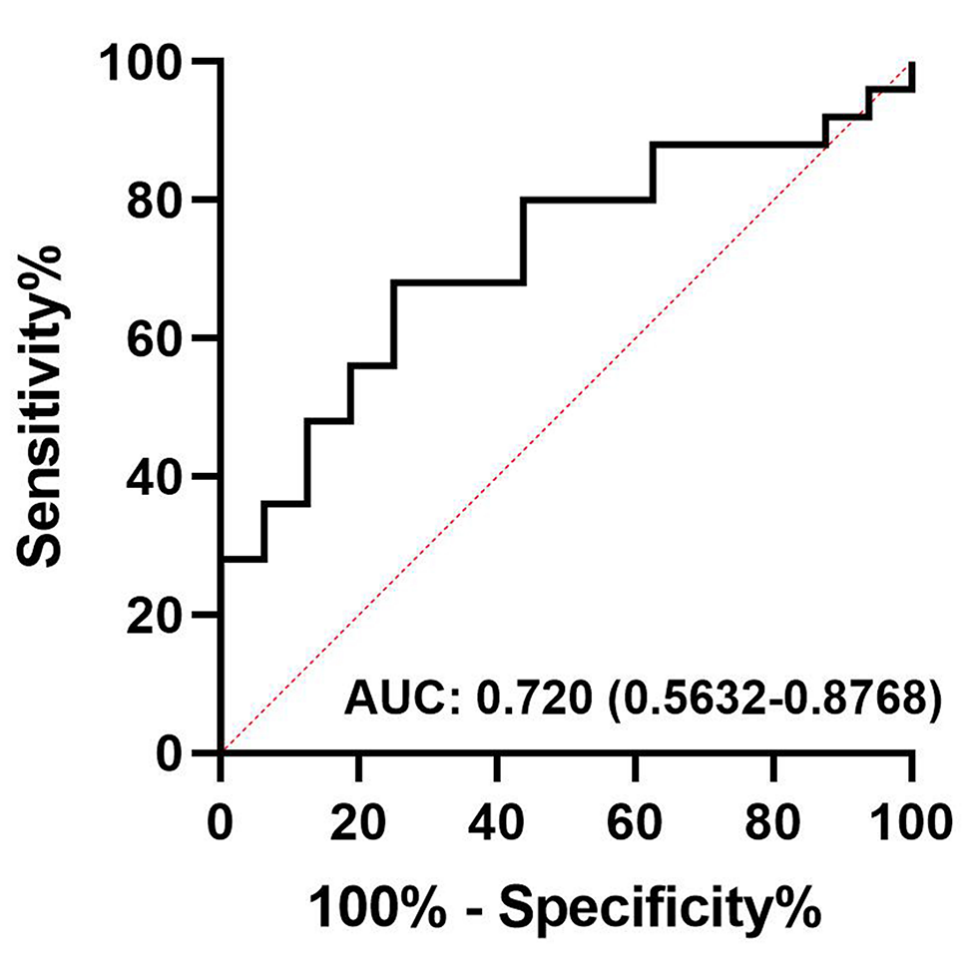
Receiver operating characteristic (ROC) model. The ROC based on *Bifidobacterium longum* at T0 predict infectious complications during chemotherapy with the area under the curve (AUC) of 0.720 (95% CI: 0.5632–0.8768, *p* = 0.0187)

To investigate whether the relative abundances of species at baseline could predict infectious complications during induction chemotherapy, multiple univariate analyses were performed on the top 20 species with the highest abundance, and the ROC curve was used to evaluate the performance of prediction model (Supplementary Table 1). We found that *Bifidobacterium Longum* achieved the highest AUC value, with an area of 0.720 (95% CI: 0.5632–0.8768, *p* = 0.0187) (Fig. 4).

## Discussion

The short-term dynamic shifts in gut microbiota and its functional features in children with ALL during induction chemotherapy and the microbial predictive markers for infection are largely unknown, especially in paediatric populations. In the current study, we applied species-level metagenomic sequencing and revealed a remarkably changing gut microbiota profile in children with ALL undergoing induction chemotherapy. We also found that the abundance of *Bifidobacterium longum* prior to commencement of chemotherapy could predict the occurrence of infection during chemotherapy.

Chemotherapeutic treatments can greatly influence the composition of gut microbiota, but not in an advantageous manner [17]. Indeed, the potent anticancer drugs can induce severe atrophy of intestinal villi and collapse of muscularis mucosa, promoting a series of inflammatory responses in the gut [18]. Moreover, the therapeutic drugs can change the gut microbiota by affecting the release of bile acids and secondary metabolic process [19]. In our study, the most evident changes in gut microbiota during chemotherapy were sharp decreases in microbial richness and evenness (to a lesser extent), and microbial dysbiosis, manifested in decreased abundances in beneficial microbes such as *Bifidobacterium longum* and *Akkermansia muciniphila*, and increased abundances of pathogens such as *Escherichia coli* and *Klebsiella pneumoniae*. A previous study compared the gut microbiome between paediatric patients with ALL at the time of diagnosis and healthy individuals, it was found that the DNA content of stool of ALL patients was 29.6% lower than that of healthy controls [20]. Another study also found that bacterial α-diversity was lower in children with newly-diagnosed ALL than their siblings [9]. In a study conducted by Thomas et al., the α- and β-diversity were not statistically different between ALL survivors (who had a history of ALL, at least one year after completion of therapy for ALL) and their siblings [21]. However, another study conducted by Bhuta et al. compared the gut microbiota of nine paediatric ALL survivors (the median time from end of therapy to stool collection was 24 months) and ten healthy sibling controls, they found that the Pielou’s evenness of gut microbiota in survivors of ALL was significantly lower than that of their healthy siblings, and the composition of gut microbiota differed between the two groups, suggesting a durable impact of ALL therapy lasting for years after completion of treatment [22]. Another study also supports the finding that the α-diversity of gut microbiota is decreased in survivors of childhood ALL [23]. In the present study, we showed a continuously decrease in microbial richness and evenness of gut microbiota during chemotherapy. In most instances, a higher microbial diversity has been linked with a relatively “healthier” microbial ecosystem, because it indicates higher probability of presence of functionally interconnected species that can either collaborate by sharing functions and metabolites required for operating specific functionality, or compensate for the absence of missing species when there is a disturbance [24]. 

The search for gut microbiota changes in ALL patients prior, during and after chemotherapy has yielded contrasting results. Some studies found a relatively stable gut microbiota structure during or after chemotherapy [25–27], while other studies suggested otherwise [11, 20, 28–30]. As it usually takes a long period of time for a ALL patient to complete the course of chemotherapy, the gut microbiota are influenced by various factors during the process, which would give less weight to the disease factors. The survival rates of ALL have exceeded 90% [31], but infectious complication remains an urgent issue that affects the quality of life and long-term prognosis. Therefore, we believe that a short-term observation of changes in gut microbiota during chemotherapy is of important clinical significance. We found a sharp shift in gut microbiota during the first month of chemotherapy, a change greater than that seen in long-term chemotherapy. However, it is important to note that the changes in gut microbiota during chemotherapy are not only influenced by chemotherapeutic drugs, other factors, such as dietary, age, antibiotic use, surrounding environment, and indirect effects exerted by chemotherapeutic drugs such as immunosuppression are also noteworthy. In the present study, the faecal samples were collected during the time when the patients were hospitalized and provided with standardized food recommendation. Therefore, it minimized the influence from diet and environment. Notably, none infectious complications were observed within the first week of chemotherapy, suggesting relatively competent immune function of patients at the beginning of induction chemotherapy. In a study conducted by Montassier et al., researchers found that the abundance of *Proteobacteria* was significantly increased after chemotherapy without using antibiotics [19]. Consistent with previous studies, we observed a continuously increasing abundance of *Proteobacteria* during chemotherapy. *Proteobacteria* is known as a microbial signature of gut microbiota dysbiosis [32]. A possible mechanism underlying the expansion of *Proteobacteria* is that when excessive inflammation occurs in the intestine, the facultative anaerobic *Proteobacteria* can take advantage of the situation through aerobic respiration and proliferate [33]. The baseline *Proteobacteria* has also been used to predict febrile neutropenia during the induction and reinduction I phases of chemotherapy [28]. In a study conducted by Chua et al., the abundance of *Bacteroidetes* decreased significantly upon the commencement of chemotherapy [11]. However, we did not observe such trend of *Bacteroidetes* in our study. This inconsistency may be attributed to sample types (anal swab sample versus faecal sample), sequencing methods (16 S ribosomal RNA versus metagenomic shotgun sequencing) and regional differences.

In the current study, we identified *Bifidobacterium longum* as the key species that could predict infectious complications during chemotherapy, which might provide a potential strategy for microbiota-targeted intervention. *Bifidobacterium* species are commensal gut microbiota that has been widely recognized to exert plentiful beneficial effects on the host. Emerging evidence has shed light on their mechanism of action, including producing bioactive substances, such as polysaccharides, short-chain fatty acids, and serine protease inhibitors, and modulating immune responses from its intestinal niche [34]. In addition to its broad benefits, *Bifidobacterium* spp. has also been found to have anti-cancer effect by promoting mitochondrial-mediated apoptosis and inhibiting growth factor signalling of cancer cell. Furthermore, they can reduce adverse effects of chemotherapy by inhibiting proinflammatory cytokines and reducing chemotherapy-induced mucositis and diarrhea [35]. Owing to their beneficial effects, the *Bifidobacterium* spp. have been widely used as probiotics. A randomized controlled trial conducted by Wada et al. discovered that administration of *Bifidobacterium breve* could effectively prevent infection in patients undergoing chemotherapy [36]. They also found that *Bifidobacterium breve* supplementation promoted growth of anaerobes and decreased population levels of *Enterobacteriaceae* [36]. Based on the findings of our study, future studies aiming to investigate the value of *Bifidobacterium longum* supplementation on prevention of infection in children with ALL during chemotherapy, as well as its mechanism of action, are warranted. However, it should be noted that although statistically significant, *Bifidobacterium longum* is insufficient for discrimination and prediction in the clinical setting.

Based on functional annotations, the pathway of pyruvate fermentation to acetate and lactate was downregulated at T2. It has been revealed that gut microbiota-derived acetate not only facilitates the production of immunoglobin (Ig)A, but directs specific IgA binding to certain bacteria such as Enterobacterales [37]. Moreover, the assimilatory sulfate reduction pathway was elevated at T2. Sulfate-reducing bacteria metabolizes sulfate to hydrogen sulfide (H_2_S), which exerts toxic effects on the intestine and is associated with inflammatory bowel disease. For example, butyrate is the major energy source for colonocytes, and H_2_S disrupts butyrate oxidation in colonocytes [38]. Assimilatory sulfate reduction pathway uses sulfate to produce amino acid cysteine [39], suggesting mechanism of compensation.

We acknowledge several limitations in the current study. First, antibiotics treatments prior to or during chemotherapy may influence interpretation of results. Empiric treatment of fever with broad spectrum antibiotics before specific pathogens were identified could skew the changes in gut microbiota composition during chemotherapy. In the current study, 59% of patients were diagnosed with infection and were treated with antibiotics. Thus, there is an unmeasured effect of antibiotic use on the microbial composition over time. Second, our previous study indicated regional variation in the gut microbiota [40], but we did not stratify patients into different regions due to a small sample size. Third, a more quantitative measure of specific gut microbiota abundance should be performed to more definitively conclude that the absolute abundance of *Bifidobacterium longum* was quantitatively different. Furthermore, the AUC values of species were not satisfying, a larger sample size is warranted to further explore the microbial biomarkers for complications during chemotherapy.

## Conclusions

Overall, our study reveals the dynamic changes in gut microbiota and its functional pathways in children with ALL during induction chemotherapy. Moreover, we found that the ROC models based on *Bifidobacterium longum* at T0 calculated an AUC of 0.720. For the first time, we describe the short-term dynamics of gut microbiota and its functional pathways in children with ALL during induction chemotherapy, a phase when paediatric ALL patients are most susceptible to infection and clinically relevant.

### Electronic supplementary material

Below is the link to the electronic supplementary material.

Supplementary Material 1

## Data Availability

No datasets were generated or analysed during the current study.
