# Supplementary material for: Microbial metagenomic shifts in children with acute lymphoblastic leukaemia during induction therapy and predictive biomarkers for infection
Source: Ann Clin Microbiol Antimicrob. 2024 Jun 15;23:52. doi: 10.1186/s12941-024-00717-z (PMC11180392; doi:10.1186/s12941-024-00717-z)
Supplement: Supplementary file 1 — Supplementary Material 1 [file 12941_2024_717_MOESM1_ESM.docx]

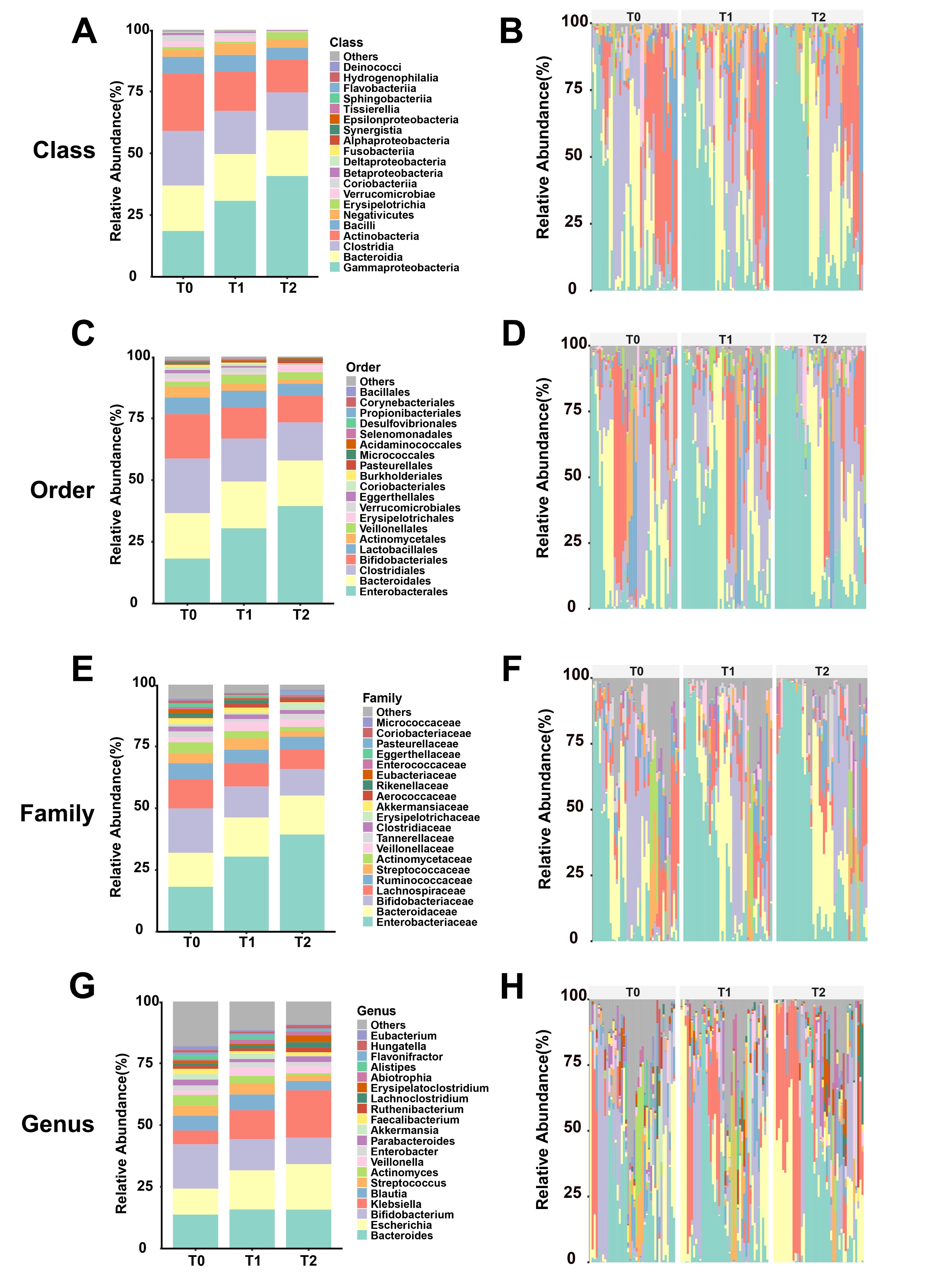


Fig. S1: Relative abundances of gut microbiota in children with ALL during induction chemotherapy at (A-B) class, (C-D) order, (E-F) family, and (G-H) Genus levels.


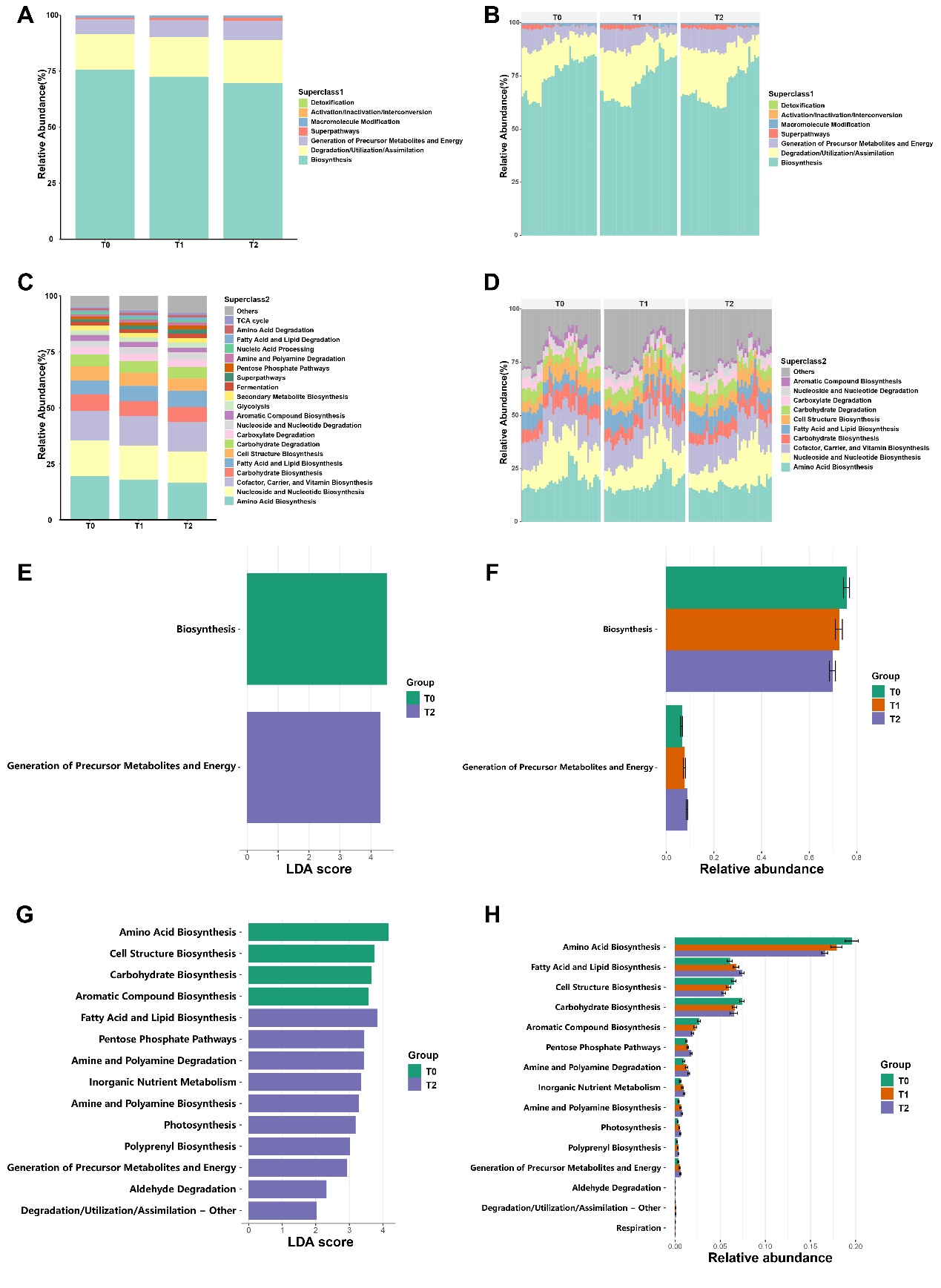


Fig. S2: The microbial functional pathways change significantly during induction chemotherapy. (A-B) Relative abundances of microbial functional pathways at the three timepoints based on superclass 1, clustered in groups (A) or individually (B). (C-D) Relative abundances of microbial functional pathways at the three timepoints based on superclass 2, clustered in groups (C) or individually (D). (E) The microbial functional pathways of superclass 1 that are significantly different between the three timepoints based on LEfSe analysis. (F) The relative abundances of microbial functional pathways of superclass 1 that are significantly different between the three timepoints. (G) The microbial functional pathways of superclass 2 that are significantly different between the three timepoints based on LEfSe analysis. (H) The relative abundances of microbial functional pathways of superclass 2 that are significantly different between the three timepoints. Data are represented as mean ± SD.

| Species | Area | 95% CI | P value |
| --- | --- | --- | --- |
| *Escherichia coli* | 0.5288 | 0.3481-0.7094 | 0.7586 |
| *Klebsiella pneumoniae* | 0.5513 | 0.3669-0.7356 | 0.5838 |
| *Bifidobacterium longum*  *Bacteroides vulgatus*  *Ruminococcus gnavus*  *Bifidobacterium breve*  *Bacteroides fragilis*  *Bifidobacterium pseudocatenulatum*  *Veillonella parvula*  *Enterobacter cloacae complex*  *Klebsiella variicola*  *Bacteroides uniformis*  *Akkermansia muciniphila*  *Faecalibacterium prausnitzii*  *Ruthenibacterium lactatiformans*  *Parabacteroides distasonis*  *Klebsiella quasipneumoniae*  *Klebsiella aerogenes*  *Flavonifractor plautii* | 0.7200  0.5500  0.6400  0.5325  0.5025  0.6025  0.6150  0.5875  0.5325  0.5688  0.5800  0.6225  0.5050  0.5425  0.5238  0.5538  0.5363 | 0.5632-0.8768  0.3733-0.7267  0.4582-0.8218  0.3452-0.7198  0.3176-0.6874  0.4176-0.7874  0.4339-0.7961  0.4103-0.7647  0.3521-0.7129  0.3847-0.7528  0.3989-0.7611  0.4512-0.7938  0.3122-0.6978  0.3552-0.7298  0.3369-0.7106  0.3684-0.7391  0.3530-0.7195 | 0.0187*  0.5930  0.1345  0.7283  0.9787  0.2732  0.2189  0.3496  0.7283  0.4624  0.3924  0.1903  0.9574  0.6496  0.7996  0.5656  0.6984 |
| *Hungatella hathewayi* | 0.5800 | 0.4001-0.7599 | 0.3924 |

***Table S1:* Performance of prediction model.**
